# Selection of New Appropriate Reference Genes for RT-qPCR Analysis *via* Transcriptome Sequencing of Cynomolgus Monkeys (*Macaca fascicularis*)

**DOI:** 10.1371/journal.pone.0060758

**Published:** 2013-04-15

**Authors:** Sang-Je Park, Young-Hyun Kim, Jae-Won Huh, Sang-Rae Lee, Sang-Hyun Kim, Sun-Uk Kim, Ji-Su Kim, Kang-Jin Jeong, Kyoung-Min Kim, Heui-Soo Kim, Kyu-Tae Chang

**Affiliations:** 1 National Primate Research Center, Korea Research Institute of Bioscience and Biotechnology, Chungbuk, Republic of Korea; 2 Department of Biological Sciences, College of Natural Sciences, Pusan National University, Busan, Republic of Korea; 3 University of Science & Technology, National Primate Research Center, Korea Research Institute of Bioscience and Biotechnology, Chungbuk, Republic of Korea; Oregon Health & Science University, United States of America

## Abstract

In the investigation of the expression levels of target genes, reverse transcription quantitative real-time polymerase chain reaction (RT-qPCR) is the most accurate and widely used method. However, a normalization step is a prerequisite to obtain accurate quantification results from RT-qPCR data. Therefore, many studies regarding the selection of reference genes have been carried out. Recently, these studies have involved large-scale gene analysis methods such as microarray and next generation sequencing. In our previous studies, we analyzed large amounts of transcriptome data from the cynomolgus monkey. Using a modification of this large-scale transcriptome sequencing dataset, we selected and compared 12 novel candidate reference genes (*ARFGAP2*, *ARL1*, *BMI1*, *CASC3*, *DDX3X*, *MRFAP1*, *ORMDL1*, *RSL24D1*, *SAR1A*, *USP22*, *ZC3H11A*, and *ZRANB2*) and 4 traditionally used reference genes (*ACTB*, *GAPDH*, *RPS19*, and *YWHAZ*) in 13 different whole-body tissues by the 3 well-known programs geNorm, NormFinder, and BestKeeper. Combined analysis by these 3 programs showed that *ADP-ribosylation factor GTPase activating protein 2* (*ARFGAP2*), *morf4 family associated protein 1* (*MRFAP1*), and *ADP-ribosylation factor-like 1* (*ARL1*) are the most appropriate reference genes for accurate normalization. Interestingly, 4 traditionally used reference genes were the least stably expressed in this study. For this reason, selection of appropriate reference genes is vitally important, and large-scale analysis is a good method for finding new candidate reference genes. Our results could provide reliable reference gene lists for future studies on the expression of various target genes in the cynomolgus monkey.

## Introduction

Reverse transcription quantitative real-time polymerase chain reaction (RT-qPCR) is a widely used experimental method for the measurement of mRNA expression levels, because this method has several advantages such as specificity, accuracy, and cost-effectiveness. Gene expression data obtained using RT-qPCR can be affected by a number of parameters such as differing sample amounts, RNA quality, purity, enzymatic efficiency in reverse transcription, and PCR efficiency [1], [2]. Because of these reasons, normalization to the level of a constitutively expressed gene, termed the reference gene or the internal control gene, is used. In numerous studies, traditional reference genes such as *glyceraldehyde-3-phosphate dehydrogenase* (*GAPDH*) and *β-actin* (*ACTB*) are frequently used for normalization. However, these genes have been shown to have variable expression levels across tissue types and experimental conditions [1], [3]. Therefore, various studies have focused on the selection of suitable reference genes among the several candidate reference genes in specific tissue types and experimental conditions. Recently, studies have focused on the selection of new reference genes in various species such as human, rhesus monkey, dog, rat, *Escherichia coli*, and buckwheat using microarray and transcriptome sequencing analyses [4]–[9]. These results showed that the expression levels of traditionally used reference genes such as *GAPDH*, *ACTB*, and *beta-2-microglobulin* (*B2M*) are unstable, and new reference genes identified from microarray and transcriptome sequencing analyses showed high stability in different experimental species and conditions. Therefore, the selection of new reference genes using microarray and transcriptome sequencing data could provide more reliable and appropriate reference genes for the normalization of target gene expression levels.

The cynomolgus monkey (*Macaca fascicularis*) is one of the most commonly used nonhuman primate animal models for studying various diseases, such as inflammation, atherogenesis, chronic wasting disease (CWD), amyotrophic lateral sclerosis (ALS), Parkinson’s disease, Alzheimer’s disease, and chikungunya disease [10]–[15] because of its biological and behavioral similarities to, and close genetic relationship with, humans [16]. Among the genus *Macaca*, the rhesus monkey (*Macaca mulatta*) is more widely used than the cynomolgus monkey for biomedical research [17]. However, studies using rhesus monkeys were restricted due to an export ban of the rhesus monkey from India in 1977. This restriction led to the increased use of the cynomolgus monkey in various studies [18]. Moreover, the cynomolgus monkey has several advantages over the rhesus monkey, including easy handling due to its smaller body size and weight, low cost and ease of availability, and lack of seasonal fertility [19], [20]. Moreover, researchers have access to more abundant gene information *via* numerous full-length mRNA sequences than in the case of rhesus macaques [20]–[26]. Therefore, the cynomolgus monkey could be a valuable experimental nonhuman primate animal model for various studies.

The aim of this study was to select appropriate reference genes and compare the newly identified 12 candidate reference genes from our previous transcriptome sequencing data [20] with 4 traditionally used reference genes for the normalization procedure in the cynomolgus monkey. The stabilities of *ADP-ribosylation factor GTPase activating protein 2* (*ARFGAP2*), *ADP-ribosylation factor-like 1* (*ARL1*), *BMI1 polycomb ring finger oncogene* (*BMI1*), *cancer susceptibility candidate 3* (*CASC3*), *DEAD (Asp-Glu-Ala-Asp) box polypeptide 3, X-linked* (*DDX3X*), *morf4 family-associated protein 1* (*MRFAP1*), *ORM1-like 1* (*Sachharomyces cerevisiae*) (*ORMDL1*), *ribosomal L24 domain containing 1* (*RSL24D1*), *SAR1 homolog A* (*S. cerevisiae*) (*SAR1A*), *ubiquitin specific peptidase 22* (*USP22*), *zinc finger CCCH-type containing 11A* (*ZC3H11A*), *zinc finger, RAN-binding domain containing 2* (*ZRANB2*), *ACTB, GAPDH, ribosomal protein S19* (*RPS19*), and *tyrosine 3-monooxygenase/tryptophan 5-monooxygenase activation protein ζ polypeptide* (*YWHAZ*) were analyzed using the geNorm [1], NormFinder [27], and BestKeeper [28] software programs in 13 different body tissues of the cynomolgus monkey.

## Materials and Methods

### Ethics Statement

All animal procedures and study design were conducted in accordance with the Guidelines of the Institutional Animal Care and Use Committee (KRIBB-AEC-11010) in the Korea Research Institute of Bioscience and Biotechnology (KRIBB).

### Specific Pathogen-free Crab-eating Macaques

Adult female (6 years old) cynomolgus monkey (*Macaca fascicularis*), origin is Vietnam and imported from China with the Convention on International Trade in Endangered Species of Wild Fauna and Flora (CITES) permit, weighing about 5 kg, were used. All animals were provided by the National Primate Research Center (NPRC) of Korea. In our experiments, specific pathogen-free (SPF) animals were used. All animals underwent a complete physical, viral, bacterial, and parasite examination. On physical examination, SPF animals were examined using various criteria, including coat condition, appearance, weight, sex, and date of birth. An enzyme immunoassay was performed to detect viruses such as simian herpes B virus (BV); simian T-cell lymphotropic/leukemia virus (STLV)-1 and -2; simian immunodeficiency virus (SIV); simian retrovirus (SRV)-1, -2, and −5; and simian varicella virus (SVV). In addition, tests were performed to detect *Mycobacterium tuberculosis* (TB), *Shigella* spp., *Salmonella* spp., and *Yersinia* spp. For the TB skin test, all animals were tested by an intradermal injection in the eyelid, and the remaining bacterial examination items were checked by fecal culture tests. In our SPF animals, all items in the above tests were negative. The monkeys were kept indoors in individual cages and fed commercial monkey chow2 (Harlan, Houston, TX) supplemented daily with various fruits, and supplied water *ad libitum*. Environmental conditions were controlled to provide a temperature of 24°C ±2°C, a relative humidity of 50±5%, 100% fresh air at a rate of ≥12 room changes per hour, and a 12∶12 h light:dark cycle. The monkey was given access to environmental enrichment such as approved toys, perches, or music to promote psychological well-being. Their health was monitored by the attending veterinarian consistent with the recommendations of the Weatherall Report.

The most important issue for transcriptome sequencing is the preparation of fresh and healthy tissue samples. Therefore, 13 tissues, such as the cecum, cerebrum, heart, kidney, liver, lung, pancreas, salivary gland, skeletal muscle, small intestine, spleen, stomach, and uterus, were obtained from SPF female adult cynomolgus monkeys following deep anesthesia using ketamine (20 mg/kg) by intramuscular injection, and perfusion with diethylpyrocarbonate (DEPC)-treated cold phosphate-buffered saline (PBS) was conducted via the common carotid artery with RNase inhibitors to inhibit blood contamination and promote the recovery of intact RNA molecules from the tissue samples.

### Total RNA Isolation and cDNA Preparation

Total RNA was extracted from the 13 different whole-body tissues using the RNeasy Mini kit (Qiagen, Valencia, CA) according to the manufacturer’s instructions. The RNase-free DNase kit (Qiagen) was used to eradicate DNA contamination from the total RNA preparations. Total RNA was quantified using a NanoDrop® ND-1000 UV-Vis Spectrophotometer. Moloney-Murine Leukemia Virus (M-MLV) reverse transcriptase was used for the reverse transcription reaction in the presence of an RNase inhibitor (Promega, Madison, WI), with an annealing temperature of 42°C. We performed PCR amplification without the reverse transcription (RT) reaction using pure RNA samples (no-RT control), and determined that the prepared mRNA samples did not contain genomic DNA.

### Primer Design and Standard Curve Analysis

For the development of specific primers for the 16 candidate reference genes, primer pairs were designed using the Primer3 program (http://frodo.wi.mit.edu/primer3/; Table 1) [29]. The gene sequences were obtained from our previous study of the sequencing the transcriptome of the cynomolgus monkey [20]. BLAST searches were performed to confirm the gene specificity of the primer sequences, and the results showed the absence of multi-locus matching at individual primer sites. Most primers spanned at least 2 exons or have a large intron sequence between the forward and reverse primers in order to avoid false-positive amplification of contaminating genomic DNA in the RNA samples. The nucleotide sequences of the RT-PCR products for the 16 candidate reference genes were obtained using standard molecular cloning and sequencing procedures (Text S1). Amplification efficiencies and correlation coefficients (R^2^ values) of the 16 genes were generated using the slopes of the standard curves obtained from serial dilutions. Standard curves with a 10-fold dilution series were used to calculate the amplification efficiency (Table 1). The amplification efficiency was calculated according to the formula: efficiency (%) = (10^(−1/slope)^ −1)×100. The efficiency range of the real-time RT-PCR amplifications for all the tested genes was 82–107%.

**Table 1:** Primers for the 16 candidate reference genes and parameters derived from RT-qPCR data analysis.

Abbreviation	Gene name	Primer* Forward(F)/Reverse(R)	Exon(s)	Amplicon size (bp)	PCR efficiency(%)	R^2^	NTC**(Cq)
*ARFGAP2*	*ADP-ribosylation factor GTPase activating protein 2*	F: GCGTCCATCTGAGCTTCATC R: CATCATTGGCTGTGCATCCA	2^nd^ 4^th^	135	89	0.98955	33.87
*ARL1*	*ADP-ribosylation factor-like 1*	F: AGACAGTTGTGACCGAGACC R: TGAGGAAGTCATGGCCTGTT	4^th^ 5^th^	136	96	0.99611	34.47
*BMI1*	*BMI1 polycomb ring finger oncogene*	F: GGCTGCTCTTTCCGGGATTT R: TACCCTCCACAAAGCACACA	1^st^ 2^nd^	105	93	0.98528	N.d.
*CASC3*	*Cancer susceptibility candidate 3*	F: CAGCCTTCTTTCCTGCAACC R: GCTGGGATGAATAGCGTTTGG	7^th^ 9^th^	139	91	0.98927	32.62
*DDX3X*	*DEAD (Asp-Glu-Ala-Asp) box polypeptide 3, X-linked*	F: GGGCGCTATATTCCTCCTCA R: ACTTCCCTCTTGAATCACTACGA	3^th^ 4^th^	139	82	0.99125	N.d.
*MRFAP1*	*Morf4 family associated protein 1*	F: GCGGATAGAGAAGAGCGAGT R: AGCCAATCTCCACCAGTTGA	1^st^ 2^nd^	82	84	0.99017	34.66
*ORMDL1*	*ORM1-like 1 (S. cerevisiae)*	F: ATTGGGAGTTGGCTTGCTTC R: TGGTCAGGAGTTTCGAAAGGT	2^nd^ 3^th^	150	96	0.99142	N.d.
*RSL24D1*	*Ribosomal L24 domain containing 1*	F: CTGGACACGGCATGATGTTC R: TCCACCTAACTTTGCGAGGA	1^st^ 2^nd^	111	107	0.99719	34.08
*SAR1A*	*SAR1 homolog A (S. cerevisiae)*	F: CCAACGCTACATCCGA/CATC R: ATCTGCACAGTCCACCAGAA	2^nd^/3^th^ 4^th^	150	104	0.99641	39.9
*USP22*	*Ubiquitin specific peptidase 22*	F: GCACAACCTGG/CCATTGATCR: AAACTTCTCTCCAGCGC/CTT	2^nd^/3^th^ 3^th^/4^th^	142	87	0.98919	33.01
*ZC3H11A*	*Zinc finger CCCH-type containing 11A*	F: AGGTTTCGGCACATGGAGAT R: TTGTGATGGAAAGCGCAGTT	3^th^ 4^th^	104	106	0.99397	N.d.
*ZRANB2*	*Zinc finger, RAN-binding domain containing 2*	F: AGTGCTAATGACTGGCAATGT R: ACCACCACCATATC/CTGTTCT	3^th^ 4^th^/5^th^	123	87	0.98756	33.3
*ACTB*	*Beta-actin*	F: ACAGAGCCTCGCCTTTGC R: CACGATGGAGGGGAAGAC	1^st^ 2^nd^	160	93	0.99031	33.99
*GADPH*	*Glyceraldehyde-3-phospate dehydrogenase*	F: ACAACAGCCTCAAGATCGTCAG R: ACTGTGGT/CATGAGTCCTTCC	6^th^ 7^th^/8^th^	112	86	0.98783	33.9
*RPS19*	*Ribosomal protein S19*	F: AGCTTGCTCCCTACGATGAG R: GACGAGCCACACTCTTGGA	3^th^ 4^th^	174	102	0.99331	32.47
*YWHAZ*	*Tyrosine 3-monooxygenase/tryptophan 5-monooxygenase activation protein, zeta polypeptide*	F: AGCAGATGGCTCGAGAATACA R: GTCATCACCAGCGGCAAC	2^nd^ 3^th^	185	96	0.98984	N.d.

* If a primer is located on 2 exons, the junctions are shown with a virgule.

** No template control.

N.d.: Not detected.

### Real-time RT-PCR Amplification

Real-time RT-PCR using SYBR Green was performed using a Rotor Gene-Q thermocycler (Qiagen). In each run, 1 µL of cDNA was used as the template for each reaction. The samples were added to 19 µL of the reaction mixture, containing 7 µL H_2_O, 10 µL Rotor Gene SYBR Green PCR mastermix (Qiagen), and 1 µL each of the forward and reverse primers. Real-time RT-PCR amplification of the 16 genes was performed in 40 cycles of 94°C for 5 sec and 60°C for 10 sec. The amplification specificity of each RT-qPCR assay was confirmed by melting curve analysis. The temperature range for the analysis of the melting curves was 55°C–99°C for 5 sec. As shown in Figure S1, each primer pair showed a single, sharp peak, thereby indicating that the primers amplified a single specific PCR product. The quantification cycle (Cq) values of the 16 candidate reference genes were obtained from the take-off point. Amplification was not detected in the no-template controls (NTC) for *BMI1*, *DDX3X*, *ORMDL1*, *ZC3H11A*, and *YWHAZ*, and was detected only after 32 cycles for the other 11 genes (Table 1). Therefore, minute amounts of primer dimers did not affect the fluorescence level of the amplified target gene. These experiments were performed in triplicate, at the least.

### Characterization of the Analysis Programs

The geNorm program [1] provides a measure of gene expression stability (M value), which is the mean pair-wise variation between an individual gene and all other tested control genes. This method differs from model-based approaches, as it compares genes based on the similarity of their expression profiles. Cq values are converted to scale expression quantities using the ΔCq method and recorded in the geNorm program, which then ranks the genes based on their M values; the gene with the most stable expression has the lowest value. NormFinder [27] focuses on finding the 2 genes with the least intra- and inter-group expression variation or the most stable reference gene in intra-group expression variation. A BestKeeper [28] index is created using the geometric mean of each candidate gene’s Cq values. This index is then compared to each individual candidate reference gene by pair-wise correlation analyses, with each combination assigned a value for the Pearson correlation coefficient (r), probability (p), SD, and CV.

### Statistical Analysis

Data was analyzed using SPSS (SPSS ver. 20). One-way analysis of variance (ANOVA) with Tukey's *post hoc* tests was used to reveal significant differences.

### MIQE Guidelines

All experiments were performed according to the Minimum Information for Publication of Quantitative Real-Time PCR Experiments (MIQE) guidelines [30].

## Results

### Selection of New Candidate Reference Genes and their Expression Range

**Figure 1:**
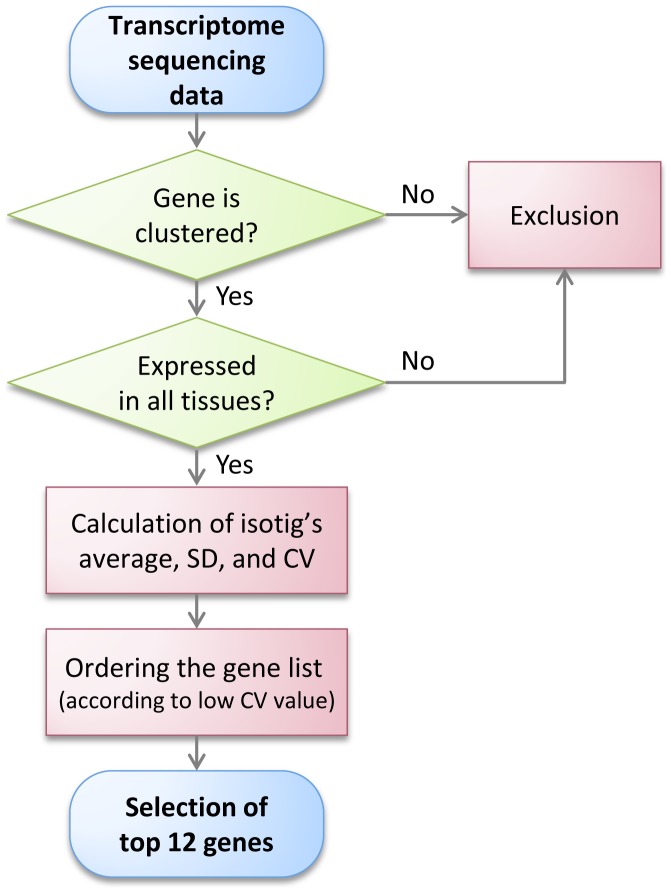
Flow chart of the selection of candidate reference genes from transcriptome sequencing data. A total of 38750 clustered genes were used for analysis. Among them, 2861 genes expressed in all tissues were identified. The average, standard deviation, and coefficient of variation (CV) of these genes were calculated. The top 12 candidate reference genes were selected based on the lowest CV value (%).

To select new candidate reference genes showing constant expression levels from our previous transcriptome sequencing data of the cynomolgus monkey [20], we analyzed the raw data of 38750 clustered genes as follows (Figure 1).

1. Sorting of genes showing expression pattern in all tested tissues.
2. Calculation of average isotig expression levels and standard deviations (SD), as well as the coefficient of variation (CV).
3. Ordering the gene list according to low CV value
4. Selection of the top 12 candidate reference genes

**Figure 2:**
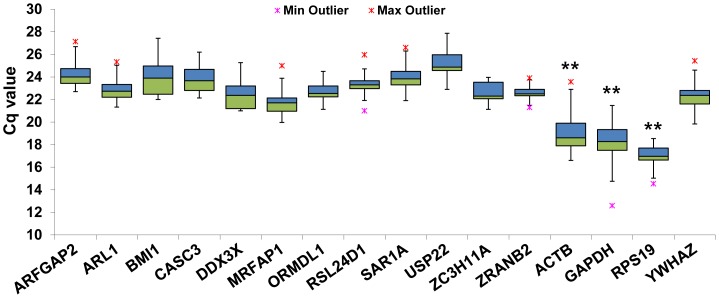
Expression levels of the 16 candidate reference genes in experimental samples. Values are given as quantification cycle (Cq) in the 13 samples. The Cq value for each reference gene is shown in terms of the median (lines), 25th to 75th percentile (boxes), and range (whiskers). *ACTB*, *GAPDH*, and *RPS19* genes were abundantly expressed compared to other 13 genes. One-way ANOVA, Tukey’s (*post hoc*) test, **p < 0.01.

According to these procedures, *BMI1* showed the least CV value, followed by *ORMDL1*, *ARL1*, *CASC3*, *ZC3H11A*, *DDX3X*, *ZRANB2*, *ARFGAP2*, *MRFAP1*, *USP22*, *RSL24D1*, and *SAR1A*. Genes with low CV values are more stably expressed than are genes with high CV values. Details of these genes such as their full names are showed in Table 1. The 16 candidate reference genes showed wide ranges of Cq values from about 13 to 28 (Figure 2). Most of the genes exhibited average Cq values ranging between 21 and 26 cycles except for *ACTB* (19 cycles), *GAPDH* (18.2 cycles), and *RPS19* (16.9 cycles). *RPS19* showed the most abundant expression pattern, with Cq values of less than 19 cycles, and *ACTB* and *GAPDH* also abundantly expressed compared to other 13 genes.

### Expression Stability of Candidate Reference Genes

We analyzed the 16 candidate genes to select the most stable and suitable reference genes using 3 well-known software programs (geNorm, NormFinder, and BestKeeper). These are freely available and generally accepted tools.

#### a) GeNorm analysis

The stability value of the 16 candidate reference genes was calculated by the geNorm program. This program selects the most suitable pair from multiple reference genes by calculating the stability values (M values) of all the tested samples. The M value is calculated as the average pair-wise variation of a particular gene compared with all other candidate reference genes. Thereafter, the gene with the highest M value is excluded, and new M values are then calculated for the remaining genes in the same manner. The genes with high M values are less stably expressed and would make bad reference genes, and those with low M values are stably expressed and would make good reference genes. The M values of the 16 candidate reference genes in the 13 different whole-body samples were calculated (Figure 2A). The reference genes *ARFGAP2* and *MRFAP1* were identified as the 2 most stable genes, with a low M-value of 0.25. These were followed by *DDX3X* (0.39), *ARL1* (0.44), *SAR1A* (0.47), *CASC3* (0.48), *BMI1* (0.51), *USP22* (0.52), *ORMDL1* (0.54), *RSL24D1* (0.56), *ZC3H11A* (0.59), *ZRANB2* (0.63), *YWHAZ* (0.69), *RPS19* (0.74), *ACTB* (0.83), and *GAPDH* (0.95) with M values in that order.

**Figure 3:**
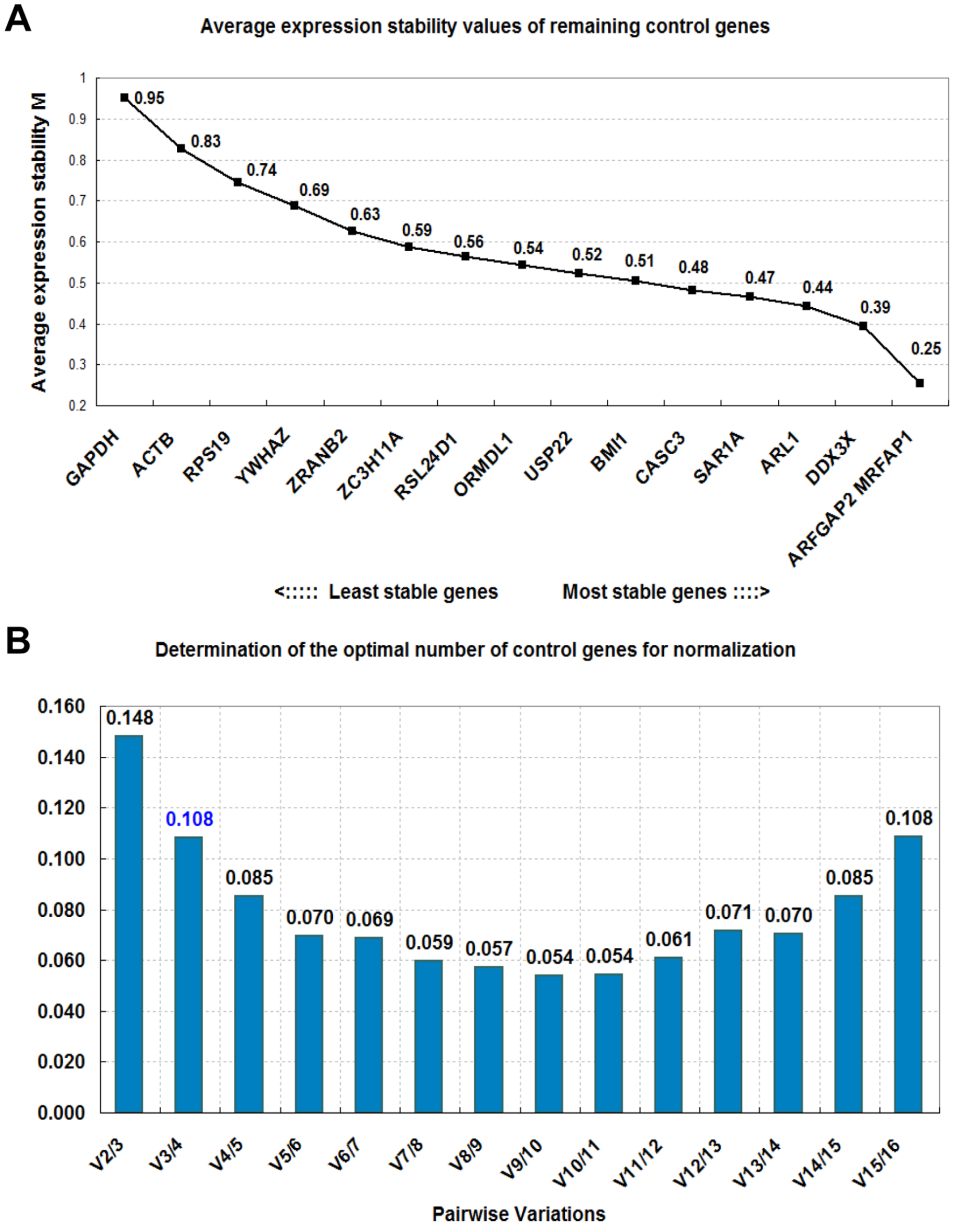
GeNorm analysis of the 13 different whole body tissues. The average expression stability (M) of 16 candidate reference genes and the best combination of 2 genes were calculated (A). Lower M values indicate more stable expression. The optimal number of reference genes for normalization was determined (B). The geNorm program calculated the normalization factor (NF) from at least 2 genes and the variable V defines the pair-wise variation between 2 sequential NF values.

According to the developers of the geNorm program, the use of a minimal number (2 or 3) of the most stable reference genes is recommended for the calculation of the normalization factor (NF) [1]. Therefore, the geNorm program can calculate the optimal number of required reference genes for obtaining reliable results from RT-qPCR studies. This calculation was performed by analysis of the pair-wise variation (V value) of sequential normalization factors (NF) with an increasing number of reference genes (NFn and NFn+1). This result was displayed in Figure 3B. The V2/3 value was 0.148 and the V value decreased consistently to V9/10. The original paper using the geNorm program proposed 0.15 as the cut-off value; implying that if the V value was <0.15, additional reference genes would be unnecessary. The pair-wise variation V2/3 was lower than 0.15; however, the V value was significantly decreased from V2/3 (0.148) to V3/4 (0.108). Therefore, in order to calculate the NF more accurately, the top 3 of the most stable reference genes were recommended in 13 tissues of the cynomolgus monkey.

#### b) NormFinder analysis

NormFinder is a tool to identify optimally stable reference genes through the determination of expression stabilities using a model-based approach in Microsoft Excel. NormFinder calculates the stability value and standard error according to the expression variation of the candidate reference genes [27]. Genes with lower stability values show less varied expression and have a stably expressed pattern and genes with higher stability values show varied expression and have the least stably expressed pattern. Analysis of our NormFinder data showed that *ARL1* (0.100) was the most stable reference gene with the lowest stability value and standard error, whereas *CASC3* (0.110), *DDX3X* (0.114), *ORMDL1* (0.114), *BMI1* (0.119), *ARFGAP2* (0.123), *MRFAP1* (0.125), *SAR1A* (0.129), *ZRANB2* (0.129), *ZC3H11A* (0.138), *RSL24D1* (0.150), *USP22* (0.153), *ACTB* (0.211), *YWHAZ* (0.211), *RPS19* (0.239), and *GAPDH* (0.299) had respectively increasing stability values (Table 2).

**Table 2:** Gene stability value calculations by NormFinder.

Gene name	Stability value	Standard error
*ARL1*	0.100	0.024
*CASC3*	0.110	0.026
*DDX3X*	0.114	0.027
*ORMDL1*	0.114	0.027
*BMI1*	0.119	0.027
*ARFGAP2*	0.123	0.028
*MRFAP1*	0.125	0.028
*SAR1A*	0.129	0.029
*ZRANB2*	0.129	0.029
*ZC3H11A*	0.138	0.031
*RSL24D1*	0.150	0.033
*USP22*	0.153	0.034
*ACTB*	0.211	0.045
*YWHAZ*	0.211	0.045
*RPS19*	0.239	0.050
*GAPDH*	0.299	0.062

#### c) BestKeeper analysis

The BestKeeper program is another Excel-based software tool, similar to geNorm and NormFinder. The program determines the most stably expressed genes based on correlation coefficient analysis for all pairs of candidate reference genes (≤10 genes), and calculates the percent coefficient of variation (CV) and standard deviation (SD) using each candidate gene’s crossing point (CP) value (the quantification cycle value; Cq) [28]. Based on these factors, the optimal reference gene for the normalization of the RT-qPCR data is determined (Table 3). This program can calculate correlation coefficients for up to 10 genes. Therefore, we selected 10 candidate genes using the results of the geNorm and NormFinder programs, and the SD values. *ACTB*, *GAPDH*, *RPS19*, and *YWHAZ* showed the lowest stability genes in these 2 programs. Thus, these 4 genes were first excluded, and then the lowest SD values of the top 10 genes were calculated using the BestKeeper program. The *ZRANB2* gene had the lowest CV (2.67) and SD (0.60) values among the 10 candidate reference genes, indicating that it was stably expressed across all tested samples. However, *ZRANB2* had a very low correlation coefficient (0.829) with the other candidate reference genes, and this means that its expression did not correlate well with the expression patterns of the other candidate reference genes. On the contrary, *DDX3X* had the highest correlation coefficient (0.982) and high CV (4.53) and SD (1.02) values. These results indicated that although its expression pattern was well correlated with other candidate reference genes, it was not stably expressed in tested samples. *ARL1* and *SAR1A* were stably expressed with high correlation coefficients, and low CV and SD values, in the tested samples.

**Table 3:** Expression stability analysis of the reference genes by BestKeeper.

Gene name	r	p-value	CV(%)	SD
*ARL1*	0.955	0.001	3.62	0.83
*SAR1A*	0.952	0.001	3.64	0.87
*ARFGAP2*	0.967	0.001	4.13	1.00
*CASC3*	0.967	0.001	4.26	1.02
*DDX3X*	0.982	0.001	4.53	1.02
*MRFAP1*	0.972	0.001	4.62	1.00
*ORMDL1*	0.942	0.001	3.32	0.75
*RSL24D1*	0.929	0.001	3.58	0.83
*ZC3H11A*	0.915	0.001	3.36	0.76
*ZRANB2*	0.829	0.001	2.67	0.60

Finally, we selected the most stable reference genes, *ARL1, ARFGAP2*, and *MRFAP1*, using the combined data from the geNorm, NormFinder, and BestKeeper programs.

## Discussion

The RT-qPCR method has been widely used and applied in gene expression analysis due to its specificity, sensitivity, and accuracy. Normalization is essential in order to obtain accurate gene expression data from RT-qPCR experiments. Therefore, a great number of studies have focused on the selection of appropriate reference genes in various species (including animals and plants) and under different experimental conditions [2]. Most gene expression studies have been performed using traditional reference genes such as *ACTB*, *GAPDH*, *B2M*, and *ribosomal protein genes* (*RPS* and *RPL*). However, in recent studies, newly identified reference genes showing more stable expression patterns than traditional reference genes have been reported by analyzing microarray and transcriptome sequencing data [8], [9]. Moreover, our results also showed that the newly identified candidate reference genes were more stably expressed than *ACTB*, *GAPDH*, *RPS19*, and *YWHAZ*. Therefore, large-scale transcriptome sequencing data could provide an excellent potential source for good candidate reference genes.

The rhesus monkey (*Macaca mulatta*) and cynomolgus monkey (*Macaca fascicularis*) are widely used animal models in the studies of various diseases. Many gene expression studies in relation to biomedical research have been performed using the RT-qPCR experiments in the cynomolgus monkey. However, to our knowledge, no studies on the selection of appropriate reference genes have been carried out in the cynomolgus monkey. In the case of the rhesus monkey, the selection of suitable reference genes has been performed in brain tissues and body tissues [7], [31]. These results have been applied in various gene expression studies using RT-qPCR experiments. To render it a more reliable and useful animal model for studying various disease-related genes, suitable reference genes must be identified in the cynomolgus monkey. Therefore, our results can be applied in many fields of study in the cynomolgus monkey.

In this study, we identified the most stable reference genes from 12 new candidate reference genes and compared these with 4 commonly used candidate reference genes in 13 different whole-body tissues of the cynomolgus monkey using the geNorm, NormFinder, and BestKeeper programs. These results showed different top-ranked reference genes between geNorm and the other 2 programs; *ARFGAP2* and *MRFAP1* in geNorm, and *ARL1* in NormFinder and BestKeeper (Table 4). This difference in the stability ranking is due to the different algorithms and analytical procedures used in these 3 programs. Therefore, the most stable reference genes for the accurate normalization of RT-qPCR data were selected through the combination of 3 programs. First, we considered the top-ranked reference genes from each programs. The best genes in the geNorm program were *ARFGAP2* and *MRFAP1*; these 2 genes ranked in the upper-middle range in the NormFinder and BestKeeper programs (Table 4). In particular, the stability values and standard error of these genes were similar to those of the top-ranked gene *ARL1* in the NormFinder program (Table 2). On the contrary, the top-ranked gene in the NormFinder and BestKeeper programs, *ARL1,* was ranked fourth from the top in the geNorm program and its M value was also similar to those of other top-ranked genes (Figure 3A). Moreover, the V value was significantly decreased at the added *ARL1* (V4/5) (Figure 3B). Therefore, normalization factor derived from the geometric means of *ARFGAP2*, *MRFAP1*, and *ARL1* could provide reliable normalization to obtain accurate gene expression data. Interestingly, commonly used traditional reference genes such as *ACTB*, *GADPH*, *RPS19*, and *YWHAZ* were ranked low in the geNorm and NormFinder programs. These results indicate that new candidate reference genes from transcriptome sequencing analysis are more accurate and trustworthy than traditional reference genes, and that the accurate selection of reference genes is essential for studies of gene expression.

**Table 4:** Ranking of candidate reference genes according to geNorm, NormFinder, and BestKeeper.

Ranking	GeNorm	Ranking	NormFinder	Ranking	BestKeeper*
1	*ARFGAP2*	1	*ARL1*	1	*ARL1*
1	*MRFAP1*	2	*CASC3*	2	*SAR1A*
3	*DDX3X*	3	*DDX3X*	3	*ARFGAP2*
4	*ARL1*	4	*ORMDL1*	4	*CASC3*
5	*SAR1A*	5	*BMI1*	5	*DDX3X*
6	*CASC3*	6	*ARFGAP2*	6	*MRFAP1*
7	*BMI1*	7	*MRFAP1*	N.R.	*ORMDL1*
8	*USP22*	8	*SAR1A*	N.R.	*RSL24D1*
9	*ORMDL1*	9	*ZRANB2*	N.R.	*ZC3H11A*
10	*RSL24D1*	10	*ZC3H11A*	N.R.	*ZRANB2*
11	*ZC3H11A*	11	*RSL24D1*	N.R.	*USP22*
12	*ZRANB2*	12	*USP22*	N.R.	*BMI1*
13	*YWHAZ*	13	*ACTB*	N.R.	*ACTB*
14	*RPS19*	14	*YWHAZ*	N.R.	*GAPDH*
15	*ACTB*	15	*RPS19*	N.R.	*RPS19*
16	*GAPDH*	16	*GAPDH*	N.R.	*YWHAZ*

* Ranking of BestKeeper program was analyzed according to genes with higher r value (above 0.950 value) and lower CV and SD values.

N.R.: Not ranked.

This study is the first to evaluate suitable reference genes from large-scale transcriptome sequencing analysis in 13 different whole-body tissues of cynomolgus monkeys, using the geNorm, NormFinder, and BestKeeper programs. Our results showed that the *ARFGAP2*, *MRFAP1*, and *ARL1* were the most appropriate reference genes. These results provide reliable information for future quantitative gene analyses in cynomolgus monkeys.

## Supporting Information

Figure S1 **Melting curve analyses of the 16 candidate reference genes from 13 different whole-body tissues.**
(TIF)

Text S1 **Nucleotide sequences of the candidate reference genes from the cynomolgus monkey.**
(DOCX)
