# Supplementary figures and images for: Selection of New Appropriate Reference Genes for RT-qPCR Analysis via Transcriptome Sequencing of Cynomolgus Monkeys (Macaca fascicularis)
Source: PLoS One. 2013 Apr 15;8(4):e60758. doi: 10.1371/journal.pone.0060758 (PMC3626658; doi:10.1371/journal.pone.0060758)

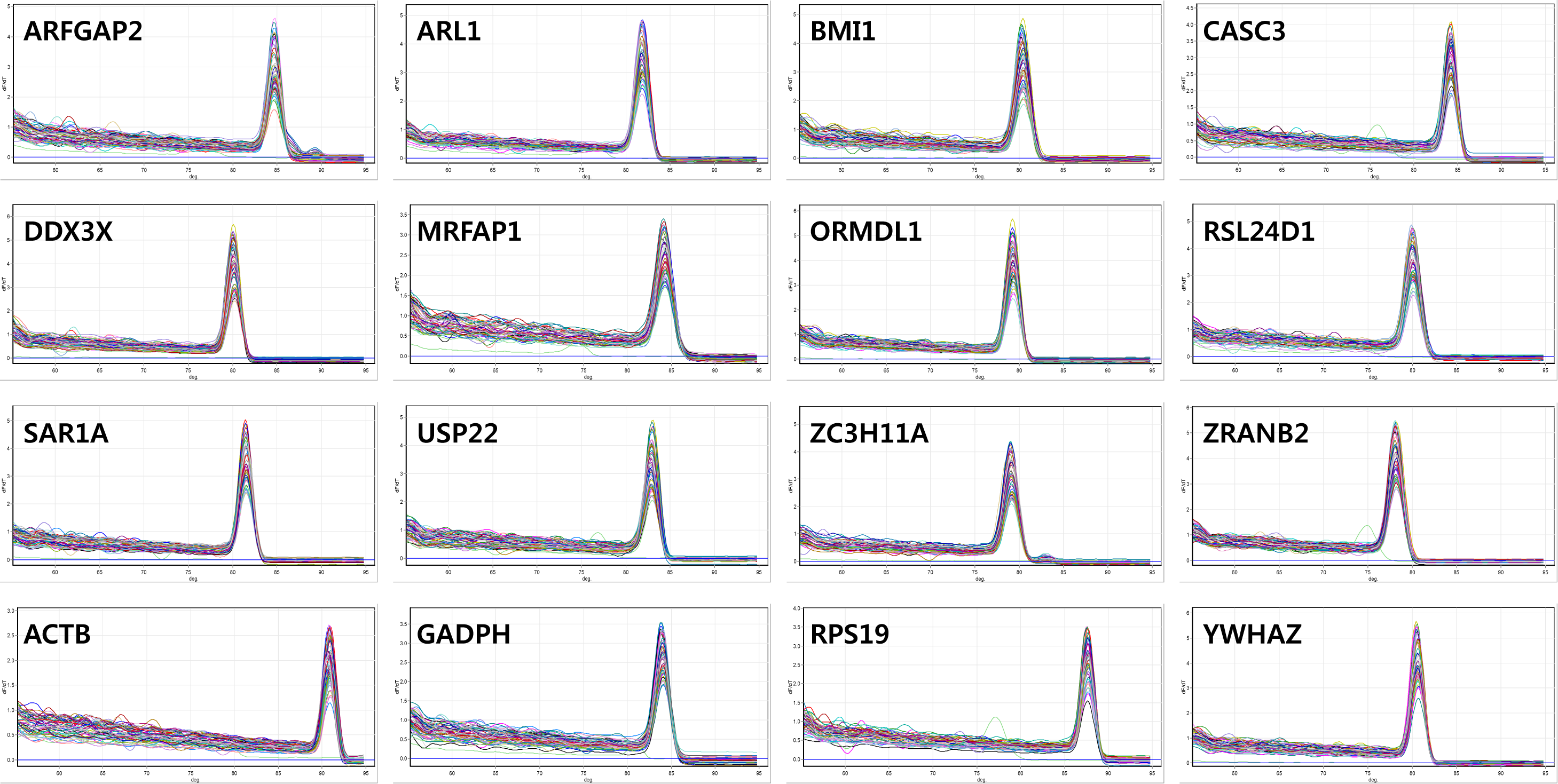

Supplement: Figure S1 — Melting curve analyses of the 16 candidate reference genes from 13 different whole-body tissues. (TIF) [file pone.0060758.s001.tif]
