# Supplementary material for: Selection of New Appropriate Reference Genes for RT-qPCR Analysis via Transcriptome Sequencing of Cynomolgus Monkeys (Macaca fascicularis)
Source: PLoS One. 2013 Apr 15;8(4):e60758. doi: 10.1371/journal.pone.0060758 (PMC3626658; doi:10.1371/journal.pone.0060758)
Supplement: Text S1 — Nucleotide sequences of the candidate reference genes from the cynomolgus monkey. (DOCX) [file pone.0060758.s002.docx]

**Text S1. Nucleotide sequences of the candidate reference genes from the cynomolgus monkey.**

>ARFGAP2

GCGTCCATCTGAGCTTCATCAGGTCCACAGAGTTGGATTCCAACTGGAACTGGTTCCAGCTGAGGTGTATGCAGGTCGGCGGGAATGCCAATGCGACGGCTTTTTTCCGCCAACATGGATGCACAGCCAATGATG

>ARL1

AGACAGTTGTGACCGAGACCGAATTGGCATTTCCAAATCGGAGTTAGTTGCCATGCTGGAGGAAGAAGAGCTGAGAAAAGCCATTTTAGTGGTGTTTGCAAATAAACAGGACATGGAACAGGCCATGACTTCCTCA

>BMI1

GGCTGCTCTTTCCGGGATTTTTTATCAAGCAGAAATGCATCGAACAACGAGAATCAAGATCACTGAGCTAAATCCCCACCTGATGTGTGTGCTTTGTGGAGGGTA

>CASC3

CAGCCTTCTTTCCTGCAACCACGGGAACTTCGAGGTATGCCCAACCATATTCACATGGGAGCAGGACCTCCACCTCAGTTTAACCGGATGGAAGAAATGGGCGTCCAGGGTGGTCGAGCCAAACGCTATTCATCCCAGC

>DDX3X

GGGCGCTATATTCCTCCTCATTTAAGGAACCGAGAAGCTACTAAAGGTTTCTACGACAAAGACAGTTCAGGGTGGAGTTCTAGCAAAGATAAGGATGCGTATAGCAGTTTTGGATCTCGTAGTGATTCAAGAGGGAAGT

>MRFAP1

GCGGATAGAGAAGAGCGAGTCGTCGTGAGCGCGGTCGGCGGTTGACAGCCAATGGATTCTGGTCAACTGGTGGAGATTGGCT

>ORMDL1

ATTGGGAGTTGGCTTGCTTCATATTGTCTTACTCAGCATTCCCTTCTTCAGTGTTCCTGTTGCTTGGACTTTAACAAATATTATACATAATCTGGGGATGTATGTATTTTTGCATGCAGTGAAAGGAACACCTTTCGAAACTCCTGACCA

>RSL24D1

CTGGACACGGCATGATGTTCGTCCGCAACGATTGCAAGGTGTTCAGATTTTGCAAATCTAAATGTCATAAAGACTTTAAAAGGAAGCGCAATCCTCGCAAAGTTAGGTGGA

>SAR1A

CCAACGCTACATCCGACATCAGAAGAGCTAACAATTGCTGGAATGACCTTTACAACTTTTGATCTTGGTGGGCATGAGCAAGCACGTCGGGTTTGGAAAAATTATCTCCCAGCAATTAATGGGATTGTCTTTCTGGTGGACTGTGCAGAT

>USP22

GCACAACCTGGCCATTGATCTGATGTACGGAGGCATCTACTGCTTTCTGTGCCAGGACTACATCTACGACAAAGACATGGAAATAATCGCCAAGGAGGAGCAGCGGAAGGCTTGGAAAATGCAAGGCGCTGGAGAGAAGTTT

>ZC3H11A

AGGTTTCGGCACATGGAGATTGATAAAAAACGCAGTGAAATTCCTTGTTATTGAGAAAATCAGCCAACGGGATGTCAAAAATTAAACTGCGCTTTCCATCACAA

>ZRANB2

AGTGCTAATGACTGGCAATGTAAAACTTGCAGCAATGTGAATTGGGCCAGAAGATCAGAGTGTAATATGTGTAATACTCCAAAGTATGCTAAATTAGAAGAAAGAACAGGATATGGTGGTGGT

>ACTB

ACAGAGCCTCGCCTTTGCCGCTCCGCCGCCCGTTCACACCCGCCGCCAGCTCGCCATGGATGATGATATCGCCGCGCTCGTTGTCGACAACGGCTCCGGCATGTGCAAGGCCGGCTTCGCGGGCGACGATGCCCCCCGGGCCGTCTTCCCCTCCATCGTG

>GADPH

ACAACAGCCTCAAGATCGTCAGCAACGCCTCCTGCACCACCAACTGCTTAGCACCCCTGGCCAAGGTCATCCATGACAACTTTGGTATCGTGGAAGGACTCATGACCACAGT

>RPS19

AGCTTGCTCCCTACGATGAGAACTGGTTCTACACACGAGCTGCTTCCACAGCGCGGCACCTGTACCTCCGGGGTGGCGCTGGGGTTGGCTCCATGACCAAGATCTATGGGGGACGTCAGAGAAACGGCGTCATGCCCAGCCACTTCAGCCGAGGCTCCAAGAGTGTGGCTCGTC

>YWHAZ

AGCAGATGGCTCGAGAATACAGAGAGAAAATTGAGACGGAGCTAAGAGATATCTGCAATGATGTACTGTCTCTTTTGGAAAAGTTCTTGATCCCCAATGCTTCACAAGCAGAGAGCAAAGTCTTCTATTTGAAAATGAAAGGAGATTACTATCGTTACTTGGCTGAGGTTGCCGCTGGTGATGAC
